# Use of Dipeptidyl-Peptidase-4 Inhibitors and the Risk of Pneumonia: A Population-Based Cohort Study

**DOI:** 10.1371/journal.pone.0139367

**Published:** 2015-10-15

**Authors:** Rogier Wvan der Zanden, Frank de Vries, Arief Lalmohamed, Johanna H. M. Driessen, Anthonius de Boer, Gernot Rohde, Cees Neef, Casper den Heijer

**Affiliations:** 1 Department of Clinical Pharmacy and Toxicology, Maastricht University, Medical Centre+, Maastricht, Netherlands; 2 Care and Public Health Research Institute (CAPHRI), Maastricht, Netherlands; 3 Division of Pharmacoepidemiology and Clinical Pharmacology, Utrecht Institute of Pharmaceutical Sciences, Utrecht, The Netherlands; Department of Clinical Pharmacy, University Medical Centre Utrecht, Utrecht, The Netherlands; 4 Department of Clinical Pharmacy, University Medical Centre Utrecht, Utrecht, Netherlands; 5 Department of Respiratory Medicine, Maastricht University, Medical Centre+, Maastricht, Netherlands; 6 Department of Sexual Health, Infectious Diseases and Environmental Health, Public Health Service South Limburg, Geleen, Netherlands; 7 Department of Microbiology, Maastricht University Medical Centre+, Maastricht, Netherlands; University of Leuven, Rega Institute, BELGIUM

## Abstract

**Background:**

Dipeptidyl-peptidase-4 inhibitors (DPP4Is) are drugs for the treatment of type 2 diabetes mellitus (T2DM). There is increasing evidence that DPP4Is may result in suppression of the immune system and may increase the risk of infections such as pneumonia. Aim of this study was to evaluate the association between the use of DPP4Is and the risk of pneumonia in a population-based study.

**Methods:**

We conducted a population-based cohort study using data from the world’s largest primary care database, the UK Clinical Practice Research Datalink (CPRD). We selected all users of non-insulin antidiabetic drugs (NIADs), including DPP4Is, between 2007 and 2012. To each NIAD user, we matched randomly selected non-users. The NIAD user’s first prescription defined the index date, which was then assigned to the matched non-users. Patients were followed from their first prescription until end of data collection or the first event of pneumonia, whichever came first. Cox regression analysis estimated the association between pneumonia and current use of DPP4Is versus 1) current use of other NIADs and 2) non-users. DPP4I use was then stratified to daily and cumulative dose. Analyses were statistically adjusted for age, sex, lifestyle factors and comorbidities and concomitant use of various other drugs.

**Results:**

Risk of pneumonia was not increased with current DPP4I use versus use of other NIADs, adjusted Hazard Ratio (HR) 0.70; 95% Confidence Interval (CI) 0.55–0.91. Also higher cumulative doses or daily doses did not further increase risk of pneumonia.

**Conclusion:**

We found no increased risk of pneumonia in T2DM patients using DPP4Is compared to T2DM patients using other NIADs. Our finding is in line with direct and indirect evidence from observational studies and RCTs. There is probably no need to avoid prescribing of DPP4Is to elderly patients who are at risk of pneumonia.

## Introduction

Dipeptidyl-peptidase-4 inhibitors (DPP4Is) (sitagliptin, saxagliptin, vildagliptin, linagliptin and alogliptin) are a new class of drugs for the treatment of type 2 diabetes mellitus (T2DM). They prolong the action of the endogenous incretin hormones glucagon-like peptide 1 (GLP-1) and glucose-dependent insulinotropic polypeptide (GIP). There is increasing evidence that DPP4Is may result in suppression of the immune system and may increase the risk of infections such as pneumonia [1,2,3,4]. Pneumonia in elderly is an important potential side effect because the risk of mortality increases with age. Its annual costs in Europe are around 10 billion euros [5]. A reduction of T-cell activity with DPP4 inhibition has been observed *in vitro* [1,2]. The (clinical) relevance of these *in vitro* studies are unclear, however. A placebo-controlled randomised clinical trial showed a dose-dependent decrease in lymphocyte count among saxagliptin users [3]. An increased risk of pneumonia among users of DPP4Is might be expected given that the risk of pneumonia is increased in diseases that are characterized by impaired T-cell function, such as the human immunodeficiency virus [6,7].

Nevertheless, conflicting results with regards to pneumonia or other (respiratory) infections as a potential side effect of DPP4Is have been reported. Summaries of product characteristics (SmPCs) of DPP4Is list infections such as (upper) respiratory tract infections as side effects [8–10]. A case-control study that used data from the World Health Organisation´s Adverse Drug Reactions database showed a 12-fold increased risk of upper respiratory tract infections with use of DPP4Is versus biguanides, whereas the risk of lower respiratory tract infections was not increased [4]. A randomized controlled trial (RCT) showed an almost 2-fold increased risk of (upper) respiratory tract infection in sitagliptin-pioglitazone users versus placebo [11]. In contrast, 3 meta-analyses of RCTs did not report elevated risks of all-cause infections with DPP4I use [12–14].

Limitations of the meta-analyses of RCTs were that most did not evaluate pneumonia, and that follow-up time was restricted. Most RCTs were designed to evaluate the efficacy of diabetes treatment, rather than detecting relatively rare infections such as pneumonia. Therefore, the aim of this study was to evaluate the association between the use of DPP4Is and the risk of pneumonia in a population-based study.

## Methods

### Data source

We used the British Clinical Practice Research Datalink (CPRD) GOLD, previously known as the General Practice Research Database (GPRD). The CPRD contains the computerised medical records of general practitioners (GPs) and holds data on 8% of the total UK population. GPs play a key role in the UK healthcare system, as they are responsible for primary healthcare and specialist referrals. Patients are affiliated with a practice that centralises the medical information from the GPs, specialist referrals, and hospitalizations. The data recorded in the CPRD include demographic information, prescription details, clinical events, preventive care provided, specialist referrals, hospital admissions, and major clinical outcomes since 1987. Studies performed with CPRD data have shown to have a high validity regarding outcomes and patient characteristics [15,16]. CPRD has been used regularly to study patients with diabetes mellitus [17–19] or pneumonia [20].

### Study population

All patients in the CPRD database of ≥18 years old with at least one prescription for non-insulin anti-diabetic drugs (NIADs) between June 2007 (the month of market introduction of DPP4Is) until 31 August 2012 were selected. The index date (i.e. date of start of follow-up) was defined by the date of the first NIAD prescription within this period. All NIAD users were matched 1:1 by sex, year of birth and general practice with non-diabetic controls. Control patients had not received any NIAD or insulin prescriptions during the entire study period and were assigned the index date of their matched NIAD user. Patients were followed-up from the index date up to the end of the study period, the patient’s transfer out of the database or the patient’s date of death, whichever came first. Patients with a history of pneumonia at baseline were excluded.

### Exposure and outcome

The follow-up time of each NIAD user was divided into intervals of current or past use based on the start and estimated end dates of NIAD or insulin prescriptions. For every prescription, a new interval was created and a patient’s person time was classified as current NIAD user. After a 3 month ‘wash out’ period from a prescriptions’ end date, a current NIAD user became past user. Past users were followed until the end of follow-up, or until a new prescription of an anti-diabetic drug was prescribed and they became a current user again. Each current NIAD user was then further stratified according to DPP4I exposure status. We used different threshold values for the definitions of current, recent and past DPP4I use because we hypothesized that the underlying hazard function with respect to onset and offset of T-cell-mediated immunosuppression would be different from discontinuation of other NIADs or insulin—which would probably reflect non-compliance or a patient being temporarily transferred out of practice. Current DPP4I users had received a DPP4I prescription in the past 2 months, for recent users this was 3–8 months ago and past DPPI4 users had received their most recent DPP4I prescription more than 8 months ago. In order to more specifically test the hypothesis of an underlying pharmacological effect, current DPP4I use was then stratified according to cumulative dose by summing the total amount of prescribed DPP4Is by using the World Health Organisation defined daily dosages (DDD) [21]. We then estimated the average daily dose by dividing the cumulative dose by the total treatment time. Both measures of exposure were expressed as sitagliptin equivalents. In order to evaluate potential confounders over time, the follow-up time of non-diabetic patients was divided into fixed time intervals of 6 months. Every patient was then followed until the end of valid data collection or the first event of pneumonia, which was identified using CPRD read codes for pneumonia. Operational definitions are added in S1 Appendix—CPRD Read codes Pneumonia.

### Potential confounders

Potential confounders that were assessed at baseline included sex, smoking status, body mass index and alcohol use. Other potential confounders were assessed at the start of each (new) NIAD prescription or period and included age, sinusitis, lung cancer, chronic obstructive pulmonary disease (COPD), human immunodeficiency virus (HIV) infection, influenza, stroke, ischaemic heart disease, dementia, the most recently recorded glycated haemoglobin (HbA1c) level in the previous year, vaccinations (influenza in previous year, *Streptococcus pneumonia* in the past 5 years and *Haemophilus influenzae* ever before) and a prescription in the previous 6 months for systemic glucocorticoids, other immunosuppressants, anticonvulsants, HIV medication, antipsychotics, proton pomp inhibitors (PPIs), H2 receptor-antagonists, bronchodilators, and inhaled corticosteroids.

### Data analysis

Cox proportional hazards regression analyses estimated adjusted hazard ratios (adj. HRs) for the risk of pneumonia in current DPP4I users compared to 1) diabetic non-users and 2) non-diabetic controls using SAS 9.2 software. Confounders were entered into the final model if they independently changed the beta coefficient for current DPP4I use by at least 5%.

### Scientific approval

This study protocol has been approved by the Independent Scientific Advisory Committee for the Medicines and Healthcare products Regulatory Agency Database Research (www.cprd.com), protocol number 13_019R. Written informed consent was not given by patients because their clinical information was de-identified and anonymized prior to analysis.

## Results

We identified 216,816 NIAD users and randomly selected the same number of non-users. After we had excluded patients with a history of pneumonia, our cohorts were comprised of 211,049 NIAD users and 212,115 non-diabetic controls. Of the NIAD users, 22,435 had used DPP4Is and 188,614 had used other NIADs and no DPP4Is at any time during follow-up. Table 1 shows the baseline characteristics. The DPP4I users were slightly younger (mean age 59 years) and more often male (57%) than NIAD users who had not been exposed to DPP4Is (61 years, 52% men) or the non-diabetic controls (61 years, 53% men). The mean follow-up time was 4.1 years for users of DPP4Is, versus 3.3 years for users of other NIADs and 3.5 years among non-diabetic controls. Regarding co-morbidities and medication use, users of DPP4Is were roughly comparable to the other NIAD users, whereas the non-diabetic controls had less co-morbidity and used less medication.

**Table 1:** Baseline characteristics.

Characteristic	DPP-4 inhibitor users (n = 22,435)		Other NIAD users(n = 188,614)		Non-diabetic controls(n = 212,115)	
Mean duration of follow-up (years, SD)	4.1	(1.5)	3.3	(1.9)	3.5	(1.8)
Number of females	9,696	43%	90,410	48%	100,543	47%
Age (years)						
Mean (SD)	58.9	(11.7)	61.2	(15.3)	60.9	(15.0)
By category						
18–49 years	4,948	22.1%	40,766	21.6%	46,300	21.8%
50–59 years	6,418	28.6%	37,148	19.7%	44,241	20.8%
60–69 years	6,787	30.3%	48,563	25.8%	55,534	26.2%
70–79 years	3,551	15.8%	42,353	22.5%	45,756	21.6%
80+ years	731	3.3%	19,784	10.5%	20,284	9.6%
BMI at index date (kg/m^2^)						
< 20.0	133	0.6%	2,525	1.3%	9,790	4.6%
20.0–24.9	1,950	8.7%	24,320	12.9%	63,180	29.8%
25.0–29.9	6,550	29.2%	60,175	31.9%	74,089	34.9%
30.0–34.9	6,811	30.4%	51,801	27.5%	30,031	14.2%
≥ 35.0	6,838	30.5%	44,719	23.7%	12,200	5.8%
Missing	153	0.7%	5,074	2.7%	22,825	10.8%
Smoking status						
Never	11,130	49.6%	94,326	50.0%	112,092	52.8%
Current	4,527	20.2%	37,353	19.8%	44,122	20.8%
Ex	6,747	30.1%	55,895	29.6%	50,199	23.7%
Missing	31	0.1%	1,040	0.6%	5,702	2.7%
Alcohol use						
No	6,312	28.1%	54,742	29.0%	38,479	18.1%
Yes	15,397	68.6%	122,770	65.1%	149,331	70.4%
Missing	726	3.2%	11,102	5.9%	24,305	11.5%
Most recent HbA1c measurement one year prior (mmol/L)						
Mean (SD)	8.3	(1.8)	7.9	(1.8)	6.3	(1.2)
By category						
≤ 6.8 mmol/L	2,685	12.0%	29,601	15.7%	4,268	2.0%
6.8–7.9 mmol/L	5,073	22.6%	36,899	19.6%	527	0.3%
> 7.9 mmol/L	6,430	28.7%	40,590	21.5%	455	0.2%
Missing	8,247	36.8%	81,524	43.2%	206,865	97.5%
Disease history						
Ischaemic heart disease	3,588	16.0%	33,748	17.9%	19,861	9.4%
Stroke	828	3.7%	10,934	5.8%	7,443	3.5%
COPD	902	4.0%	10,026	5.3%	9,143	4.3%
Sinusitis	3,194	14.2%	24,623	13.1%	29,063	13.7%
Lung cancer	14	0.1%	459	0.2%	381	0.2%
Dementia	98	0.4%	3,284	1.7%	2,441	1.2%
Drug use 6 months before						
Systemic glucocorticoids	1,021	4.6%	10,718	5.7%	8,113	3.8%
Other immunosuppressants	189	0.8%	1,802	1.0%	1,675	0.8%
Proton pump inhibitors	4,499	20.1%	38,466	20.4%	31,317	14.8%
Histamine 2-receptor antagonists	497	2.2%	4,465	2.4%	3,780	1.8%
Antipsychotics	374	1.7%	4,409	2.3%	2,808	1.3%
Anticonvulsants	753	3.4%	7,417	3.9%	5,281	2.5%
Vaccinations						
Influenza (1 year before)	13,750	61.3%	110,593	58.6%	79,661	37.6%
*Streptococcus Pneumonia* (5 years before)	7,863	35.1%	61,640	32.7%	49,329	23.3%
*Haemophilus Influenza* (ever before)	59	0.3%	921	0.5%	909	0.4%

Abbreviations:

NIAD: non-insulin anti-diabetic drugs;

SD: standard deviation;

BMI: body mass index;

HbA1c: glycated haemoglobin;

COPD: chronic obstructive pulmonary disease.

Current users of NIADs had a 1.6-fold increased risk of pneumonia, as compared with non-diabetic controls (adj. HR 1.56 (Confidence Interval [CI] 1.42–1.71). Further analyses were conducted within the cohort of NIAD users. There was no increased risk of pneumonia with current DPP4I use, as compared to other NIAD users (adj. HR 0.70; 95% CI 0.55–0.91). Discontinuation of DPP4Is did not further decrease the risk of pneumonia (adj. HRs 1.04; 95% CI 0.59–1.85 for recent users and 1.00; 95% CI 0.55–1.81 for past users). In order to more specifically test the hypothesis of an underlying pharmacological effect, we evaluated the risk of pneumonia with a higher cumulative and daily dose. However, there was no further significant dose-effect relationship between pneumonia and DPP4I use (Table 2).

**Table 2:** Use of DPP-4 inhibitors and risk of pneumonia in T2DM patients, by cumulative dose and daily dose.

Current NIAD use	No. of pneumonia events (n = 2,596) ^a^	IR (/1000 PYs)	Age/sex adjustedHR (95% CIs)	Fully adjustedHR (95% CIs)^b^
Never use of DPP4Is or GLP-1 analogues	2,252	4.0	Reference	Reference
Use of GLP-1 analogues	34	2.4	1.14 (0.81–1.61)	0.97 (0.68–1.37)
Use of DPP4Is				
Past use ^c^	11	3.5	1.07 (0.59–1.94)	1.00 (0.55–1.81)
Recent use ^c^	12	3.6	1.13 (0.64–2.01)	1.04 (0.59–1.85)
Current use ^c^	64	2.2	0.68 (0.53–0.88)	0.70 (0.55–0.91)
By cumulative dose (sitagliptin equivalents)				
Low (≤15 gram)	22	2.8	0.88 (0.58–1.34)	0.87 (0.57–1.33)
Medium (15.1–45 gram)	19	1.6	0.52 (0.33–0.81)	0.53 (0.34–0.83)
High (>45 gram)	23	2.5	0.72 (0.48–1.09)	0.77 (0.51–1.16)
By average daily dose (sitagliptin equivalents)				
One prescription only	21	3.4	1.08 (0.70–1.66)	1.06 (0.69–1.63)
Low dose (≤95mg)	14	1.7	0.54 (0.32–0.92)	0.56 (0.33–0.94)
Medium dose (95.1–105 mg)	15	1.8	0.51 (0.31–0.85)	0.56 (0.33–0.93)
High dose (>105 mg)	14	2.4	0.73 (0.43–1.24)	0.74 (0.44–1.26)

Abbreviations:

DPP4: dipeptidyl-peptidase-4;

NIAD: non-insulin anti-diabetic drug;

IR: incidence rate;

PY: patient years;

HR: hazard ratio;

GLP-1: glucagon-like peptide 1;

DDD: defined daily dose;

cum.: cumulative;.

^a^: Numbers do not add up because data on past NIAD user is not shown

^b^: Adjusted for sex, age, smoking status, BMI, alcohol use; a history of lung cancer, COPD, dementia and stroke; use of glucocorticoids, anticonvulsants, proton pump inhibitors, immunosuppressants (excluding glucocorticoids) and antipsychotics in the previous 6 months, influenza vaccination in the previous year, pneumococcal vaccination in the previous 5 years, *Haemophilus influenza* vaccination ever before, past use of NIADs and the most recently recorded HbA1c level in the previous year

^c^: Current use: a DPP4I-prescription ≤2 months before, recent use: a DPP4I-prescription 3–8 months before; past use: a DPP4I-prescription >8 months before.

Table 3 shows that the risk of pneumonia with current DPP4I use was lower in women (adj. HR 0.46; 95% CI 0.28–0.74) than in men (adj. HR 0.88; 95% CI 0.66–1.19). There was no clear trend with different age classes. Stratification of current DPP4I use to body mass index (BMI) did not disclose a trend with risk of pneumonia either: the adjusted HR was 0.77 (95% CI 0.38–1.57) for patients with normal weight (BMI 20.0–24.9), 0.87 (95% CI 0.57–1.32) for patients who were overweight (BMI 25.0–29.9), 0.59 (95% CI 0.35–0.99) for obese patients (BMI 30.0–34.9) and 0.71 (95% CI 0.43–1.16) for morbidly obese patients (BMI >35.0).

**Table 3:** Use of DPP-4 inhibitors and risk of pneumonia in T2DM patients, by sex and age.

Current NIAD use	No. of pneumonia events (n = 2,596) ^a^	IR (/1000 PYs)	Age/sex adjustedHR (95% CIs)	Fully adjustedHR (95% CIs)^b^
Never use of DPP4Is or GLP-1 analogues	2,252	4.0	Reference	Reference
Current DPP4I use^c^	64	2.2	0.68 (0.53–0.88)	0.70 (0.55–0.91)
By sex				
Male	47	2.8	0.86 (0.64–1.15)	0.88 (0.66–1.19)
Female	17	1.4	0.44 (0.27–0.71)	0.46 (0.28–0.74)
By age				
<50 years	2	0.45	0.36 (0.09–1.48)	0.36 (0.09–1.49)
50–59 years	9	1.2	0.82 (0.42–1.63)	0.90 (0.45–1.77)
60–69 years	14	1.5	0.46 (0.27–0.79)	0.50 (0.29–0.86)
70–79 years	19	3.2	0.63 (0.40–1.00)	0.64 (0.41–1.02)
≥80 years	20	13	1.15 (0.73–1.79)	1.21 (0.77–1.90)

Abbreviations:

NIAD: non-insulin anti-diabetic drug;

IR: incidence rate;

HR: hazard ratio;

DPP4: dipeptidyl-peptidase-4;

GLP-1: glucagon-like peptide 1

^a^: Numbers do not add up because data on past NIAD use, current GLP1 agonist and recent/past DPP4I is not shown

^b^: Adjusted for sex, age, smoking status, BMI, alcohol use; a history of lung cancer, COPD, dementia and stroke; use of glucocorticoids, anticonvulsants, proton pump inhibitors, immunosuppressants (excluding glucocorticoids) and antipsychotics in the previous 6 months, influenza vaccination in the previous year, pneumococcal vaccination in the previous 5 years, *Haemophilus influenza* vaccination ever before, past use of NIADs, use of GLP1 analogues, recent and past use of DPP4Is (see Table 2, footnote b) and the most recently recorded HbA1c level in the previous year

^c^: Current use: a DPP4I-prescription ≤2 months before.

## Discussion

Our study did not find an increased risk of pneumonia with current use of DPP4Is. This finding was supported by additional analyses to indirectly test our main hypothesis. Risk of pneumonia did not drop after discontinuation of DPP4Is, and a higher cumulative or daily dose did not further increase the risk. We could not detect increased risks of pneumonia in men, women, in patients with various age groups or BMI.

Our main finding of no elevated risk of pneumonia with use of DPP4Is is in line with a meta-analysis of RCTs. A pooled analysis of 25 clinical studies (n = 14,611) with sitagliptin vs. comparator (mostly placebo) in T2DM patients, showed no increased risk of pneumonia (difference in incidence rate (IR) per 100 patient years between sitagliptin and comparator 0.2 (95% CI -0.2 to 0.5) [12]. An observational study performed by Faillie and colleagues that used the same CPRD database showed that current DPP4I use was not associated with hospitalizations for community-acquired pneumonia, as compared to users of other antidiabetic drugs, adjusted odds ratio (OR) 0.80; 95% CI 0.50–1.29. Furthermore, prolonged use of DPP4Is was not associated with an increased risk of hospitalizations for community-acquired pneumonia [22], which is in line with our findings of no further increased risks with a higher cumulative dose of DPP4Is. Although Faillie and colleagues had used the same CPRD database for their research, there were several major differences in both study designs. As a result, we identified more than 4 times as many patients with pneumonia, which has added new findings rather than a replication of an earlier study [23,24]. Although we roughly included the same period of data collection (2007–2012), the source population of Faillie and colleagues was probably 50% smaller and no longer representative for the total UK population, as a result of inclusion of a subset of practices that has consented to link their data to the Hospitalisation registry of England. In addition, their outcome of interest was a hospitalization for community-acquired pneumonia, rather than an event of any pneumonia in our study. This difference has implications on the external validity of the findings: we have used a representative sample of the total UK population, whereas Faillie and collegues only studied a subset of practices in England, which have consented to link to the National Hospitalisation Registry of England. As far as we know, its representativeness for the total UK population has never been evaluated [22]. Our results are also in line with those from a case-control study that used data from the World Health Organisation´s Adverse Drug Reactions database which showed that the risk of lower respiratory tract infections was not increased among DPP4I users as compared to users of biguanides [4].

In contrast to a consistent absence of an association between DPP4I use and pneumonia or *lower* respiratory tract infections, conflicting results have been reported about the risk of all-cause infections or *upper* respiratory tract infections with DPP4I use. The same case-control study that used data from the World Health Organisation´s Adverse Drug Reactions database found a 12-fold increased risk of upper respiratory tract infections among DPP4I users versus users of biguanides [4]. It should be noted that this study was based on an adverse drug reaction database, and its ability to study causal effects is therefore limited. For example, selective over-reporting of side effects in new classes of drugs such as DPP4Is might have occurred. A Cochrane review that was comprised of 8 RCTs (n = 3,589 patients) compared sitagliptin with comparator and showed a 30% increased risk of all-cause infections (RR = 1.29 (95% CI 1.09–1.52) [25]. No differences in risk of upper respiratory tract infections and infections in general were reported in an RCT that compared DPP4I use with comparator (difference in incidence rate -0.6 (95% CI -1.7 to 0.5) for upper respiratory tract infections and 0.3 (95% CI -2.5 to 3.1) for infections in general) [12]. This is consistent with another meta-analysis of RCTs, which found no increased risk of (upper) respiratory tract infections (risk ratio 0.99; 95% CI (0.81–1.21) when the results of trials with vildagliptin and sitagliptin versus controls were pooled [26]. A systematic review and meta-analysis of 39 randomized controlled trials with DPP4Is in diabetic patients (n = 18,491, comparator mostly placebo), did not show an increased risk of all-cause infection (relative risk (RR) = 0.98 (95% CI 0.93–1.05)) or upper respiratory tract infections (RR = 0.97 (95% 0.83–1.14) [14]. Lastly, a meta-analysis of 10 studies (n = 8,719 patients) comparing DPP4Is vs. active comparators, showed no increased risk of (upper) respiratory tract infections: RR 1.00 (95% CI 0.83–1.22) [27].

Our hypothesis that DPP4I use might be associated with pneumonia was based on the observations that DPP4Is may affect T-lymphocyte function. CD26 (the proteolytically active plus inactive form) and DPP4 (the active form) have been found on B-lymphocytes, natural killer (NK) cells and specific subsets of CD4 and CD8 T-cells [28,29]. Most T-cells expressing CD26/DPP4 are part of the CD4+ population [30]. It is known that certain dysfunctions in these cells can lead to infections. For example, a low CD4 count in HIV patients makes them prone for opportunistic infections such as *Pneumocystis jirovecii* pneumonia (formerly *Pneumocystis carinii*) [31]. Although CD26/DPP4 can function as an alternative pathway for T-cell activation, it has been suggested that its enzyme activity is not conditionally required for the activation of on T-lymphocytes [29,32,33]. If T-lymphocyte activation remains possible through other pathways, this could explain our finding that DPP4I use does not lead to in increased risk of pneumonia. A small clinical study in T2DM patients who had used sitagliptin showed that median T-cell activity was not significantly different from T2DM patients who did not use sitagliptin [28]. In our cohort, unfortunately, we did not have access to intermediate endpoints such as DPP4 enzymatic activity in plasma of the patients undergoing treatment with DPP4 inhibitors or T cell counts.

A complicating factor related to our hypothesis is the possibility that DPP4Is may also possess activity for other, related enzymes, such as DPP8 and DPP9. It is suggested that DPP8/9 play a role in immune response [34]. In a human *in vitro* model, a DPP8/9 inhibitor was able to inhibit T-cell activation, whereas a selective DPP4I had no effect [2]. Finally, some DPP4Is that had an effect in certain models of immune function, have shown to also be potent inhibitors of the DPP8/9 enzymes [2]. All this could lead to the question whether DPP4 or DPP8/9 is the more important enzyme in immune function. Finally, our hypothesis is complicated by the fact that a plethora of peptides and chemokines are cleaved by DPP4 and CD26 concentrations and DPP4 activity varies between healthy subjects with different medical conditions, making it difficult to predict the effect of DPP4 inhibition on a specific outcome measure such as pneumonia [35].

Strengths of our study include its large sample size and that it is population-based, in contrast to a previous study [22]. The CPRD represents a significant number of UK residents and has proven to be a reliable source for patient characteristics and clinical diagnoses [15,16]. This allowed us to study a large, real life cohort of patients and statistically adjust for many potential confounders such as age, gender, smoking status, comorbidity and use of medication. Another strength of our study is that exposure and outcome are routinely and longitudinally collected, which minimizes the risk of information bias. Our study had a substantial mean duration of follow-up of 4.1 years among DPP4I users, which was substantially longer than most randomised clinical trials. Furthermore, we observed a 1.6-fold increased risk of pneumonia among T2DM patients versus non-diabetic controls, which indicates that the `assay sensitivity´ of our study was appropriate because these findings are consistent with existing studies that have reported positive associations between diabetes mellitus and community acquired pneumonia [36].

Our study had several limitations. A true effect may have been masked by a wide range of distortions such as residual confounding, misclassification of exposure or outcome or channelling bias. As with all observational studies, residual confounding remains a possibility. Although we were able to adjust for many possible confounders, it is possible that unmeasured confounders may have influenced our results. Misclassification of exposure can be the result of non-compliance. Misclassification of pneumonia can also have occurred, in particular if it is not being coded or diagnosed correctly or when it is diagnosed in-hospital and the GP does not (properly) code the hospital discharge letter. We considered both types of misclassification non-differential, which may have lead to a bias towards null. Theoretically, this could have masked a truly elevated risk of pneumonia. Nevertheless, we observed the opposite, i.e. DPP4I use was inversely associated with pneumonia.

Peer review has suggested that including the risk for upper and lower respiratory infections could have been an interesting additional outcome, but this was beyond the scope of the current hypothesis.

An intriguing observation was the 30% significantly decreased risk of pneumonia with DPP4I use, whereas a meta-analysis of randomized controlled trails had found no increased risk [12]. This may be explained by channelling bias in our study: new classes of drugs are often selectively prescribed certain groups of patients [37]. It is possible that in the first years after their introduction, DPP4Is were prescribed more often to more ‘health conscious’ patients actively requesting a DPP4I prescription from their physician. Random allocation of DPP4Is would eliminate such bias in RCTs.

In conclusion, we found no increased risk of infection in T2DM patients using DPP4 inhibitors compared to T2DM patients using other NIADs. Our finding is increasingly being supported by direct and indirect evidence from observational studies and RCTs. There is probably no need to avoid prescribing of DPP4Is to elderly patients who are at risk of pneumonia.
